# Post molar choriocarcinoma with solitary renal metastasis in the absence of primary uterine tumor: a case report and review of the literature

**DOI:** 10.1186/s13256-024-04464-9

**Published:** 2024-03-30

**Authors:** Mahsa Geravandi, Ali Hajihashemi, Atoosa Adibi, Reza Habibi Tirtashi

**Affiliations:** 1 Department of Radiology, Isfahan University of Medical Sciences, Isfahan, Iran; 2 Clinical Research Development Unit (CRDU), 5azar Hospital, Golestan University of Medical Sciences, Azar 6th, 5 Azar St, P.O. Box 49189-36316, Gorgan, Iran; 3 Metabolic Disorders Research Center, Golestan University of Medical Sciences, Gorgan, Iran

**Keywords:** Gestational trophoblastic disease, Hydatiform mole, Choriocarcinoma, Post molar choriocarcinoma, Renal metastasis, Spontaneous renal hemorrhage

## Abstract

**Background:**

Choriocarcinoma is a rare and highly malignant form of gestational trophoblastic disease that may develop following pregnancy, abortion, or a hydatiform mole. Renal metastatic involvement by post molar choriocarcinoma is even rarer. In this case report, we describe a unique case of post molar choriocarcinoma with a solitary renal metastasis in the absence of a primary uterine tumor and metastases in other sites, which presented with urological symptoms and spontaneous renal hemorrhage.

**Case presentation:**

A 41-year-old Persian woman with history of complete hydatiform mole presented with severe flank pain, nausea, vomiting, gross hematuria, and vaginal bleeding. Laboratory tests demonstrated a serum beta human chorionic gonadotropin hormone level of 60,000 mIU/mL. Imaging studies showed a lesion at the lower pole of the left kidney with active bleeding surrounded by hematoma, as well as an empty uterine cavity. Additionally, bilateral pleural effusion was detected without any lesion within the lungs. Subsequently, the patient underwent laparotomy, partial nephrectomy, and left para-ovarian cystectomy. Endometrial curettage was also carried out. The histopathology report revealed choriocarcinoma renal metastasis with high expression of beta human chorionic gonadotropin, cytokeratin 7, and Ki 67. Moreover, there were no malignant cells in the endometrial curettage specimens, and a corpus luteum cyst was found within the para-ovarian cyst. Further investigations revealed that the pleural effusion was free of malignant cells, and there was no evidence of metastatic lesions in the brain. As a result, the patient was referred to the oncology department to receive chemotherapy, and the beta human chorionic gonadotropin levels dropped to 5 mIU/mL after receiving courses of a standard regimen of etoposide, methotrexate, actinomycin D, cyclophosphamide, and vincristine/oncovin over 3 weeks. Finally, monthly measurements of beta human chorionic gonadotropin levels for 6 months indicated that levels have constantly remained within normal ranges, showing no evidence of recurrence or new metastasis.

**Conclusions:**

Urological symptoms such as hematuria or spontaneous renal hemorrhage might be the only presentation of post molar choriocarcinoma with renal involvement. Thus, it can be beneficial to measure serum beta human chorionic gonadotropin levels among females of childbearing age who present with unexplained urological symptoms, especially if there is a history of prior hydatiform mole.

## Background

Gestational trophoblastic disease (GTD) refers to a spectrum of infrequent pregnancy-associated tumors with aberrant proliferation of trophoblasts and the potential capability of producing beta human chorionic gonadotropin hormone (β-hCG), comprising premalignant hydatiform mole (partial and complete), as well as malignant invasive mole, choriocarcinoma, placental site trophoblastic tumor, and epithelioid trophoblastic tumor [1, 2].

Choriocarcinoma, with an incidence of 1–9.2 in 40,000 pregnancies, is a relatively rare but highly malignant type of GTD among females and might occur subsequent to a miscarriage, ectopic pregnancy, term pregnancy, or hydatiform mole [1, 3, 4]. In this regard, an antecedent molar pregnancy, particularly a complete mole, is associated with the highest risk of choriocarcinoma development [3, 5]. Choriocarcinoma is recognized for its rapid proliferation and dissemination through the bloodstream [3, 6]. According to available literature, metastatic choriocarcinoma could be found among approximately 30% of patients, and the lungs, vagina, pelvis, liver, brain, and gastrointestinal tract were the most common sites for involvement. Regarding this, with an incidence rate of 1–6.9%, renal metastasis from choriocarcinoma is extremely rare and attributed to circulatory tumoral cells disseminating from primary lung metastases [2–5, 7]. Typically, gynecological symptoms such as uterine enlargement or vaginal bleeding are identified as common presentations of choriocarcinoma. Signs of elevated hCG levels, including malaise or hyperemesis, may also accompany these symptoms [2]. However, presentations of choriocarcinoma may differ depending on the site of metastasis, for example, dyspnea, cough, and hemoptysis in pulmonary metastases or hematuria, flank pain, and renal subcapsular hematoma in renal involvement [7].

A couple of previous reports have described post molar choriocarcinoma with renal metastasis [3, 5, 8–14], manifesting a diverse range of symptoms congruent with metastases to other sites. Notably, only a few cases among them presented with pure urological symptoms [5, 8, 10]. In light of these findings, we introduce a unique case of choriocarcinoma occurring subsequent to a complete hydatiform mole with solitary renal metastasis in the absence of a primary uterine tumor and metastases to other sites that presented with pure urological symptoms, accompanied by a review of similar literature.

## Case presentation

A 41-year-old Persian woman presented to the emergency department with complaints of severe left flank pain, nausea, vomiting, gross hematuria, and vaginal bleeding. She has been experiencing intermittent left flank pain for the past 3 months, which had exacerbated over the past few days. The onset of hematuria and vaginal bleeding occurred 3 weeks previously.

**Fig. 1:**
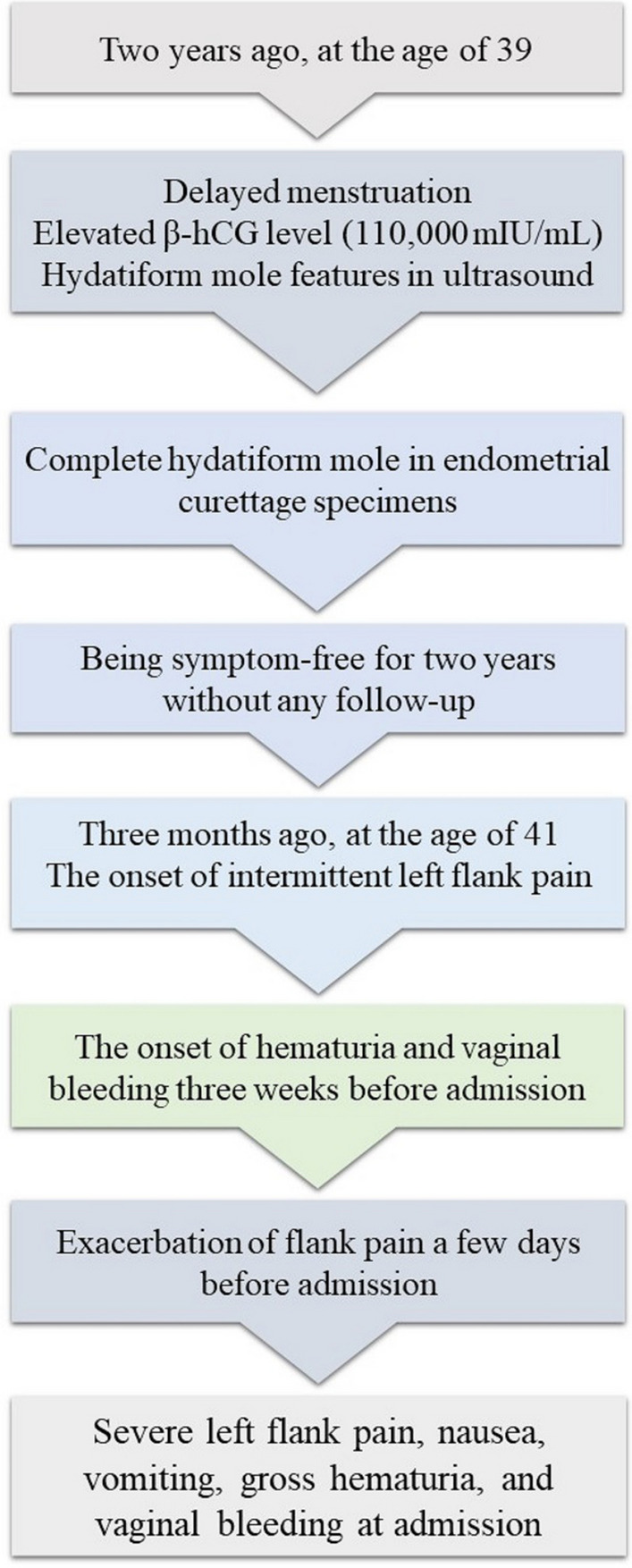
A timeline illustrating the occurrence of the patient’s previous molar pregnancy and the onset of current symptoms

In reviewing the patient’s medical history, she denied any prior history of trauma or relevant underlying medical conditions. Concerning gynecological and obstetric history, she reported a regular menstrual cycle and three pregnancies, which included an abortion, an uncomplicated full-term pregnancy terminated by a cesarean section 4 years previously, and a molar pregnancy. Two years previously, the patient experienced delayed menstruation, prompting further evaluations that revealed a serum β-hCG level of 110,000 mIU/mL, along with a suspected hydatiform mole on abdominal and transvaginal ultrasound. Consequently, she underwent endometrial curettage, and histopathological evaluation confirmed the diagnosis of a complete hydatiform mole. Although the serum β-hCG level decreased after initial treatments, the patient declined further follow-up, including regular measurements of β-hCG level. For the past 2 years, she remained uncomplicated until the onset of the symptoms mentioned above. A timeline detailing the patient’s history and symptoms is provided in Fig. 1.

Upon admission, the patient was pale and moderately distressed due to severe pain, and the following vital signs were recorded: heart rate of 95 beats/minute, respiratory rate of 24 breaths/minute, blood pressure of 110/80 mmHg, and temperature of 39 °C. Physical examination revealed unremarkable findings except for increased sensitivity on the left flank and tenderness on the left-side costovertebral angle.

Laboratory tests showed elevated levels of serum β-hCG (60,000 mIU/mL), erythrocyte sedimentation rate (40 mm/hour), and C-reactive protein (50 mg/L), as well as neutrophil-dominant leukocytosis (white blood cells 33,000 /mm^3^ and neutrophil count 29,700/mm^3^), anemia (hemoglobin 9.2 mg/dl), and hematuria (3+ blood and 50–60 red blood cells per microscopic high-power field). All other biochemical parameters were within normal limits.

**Fig. 2:**
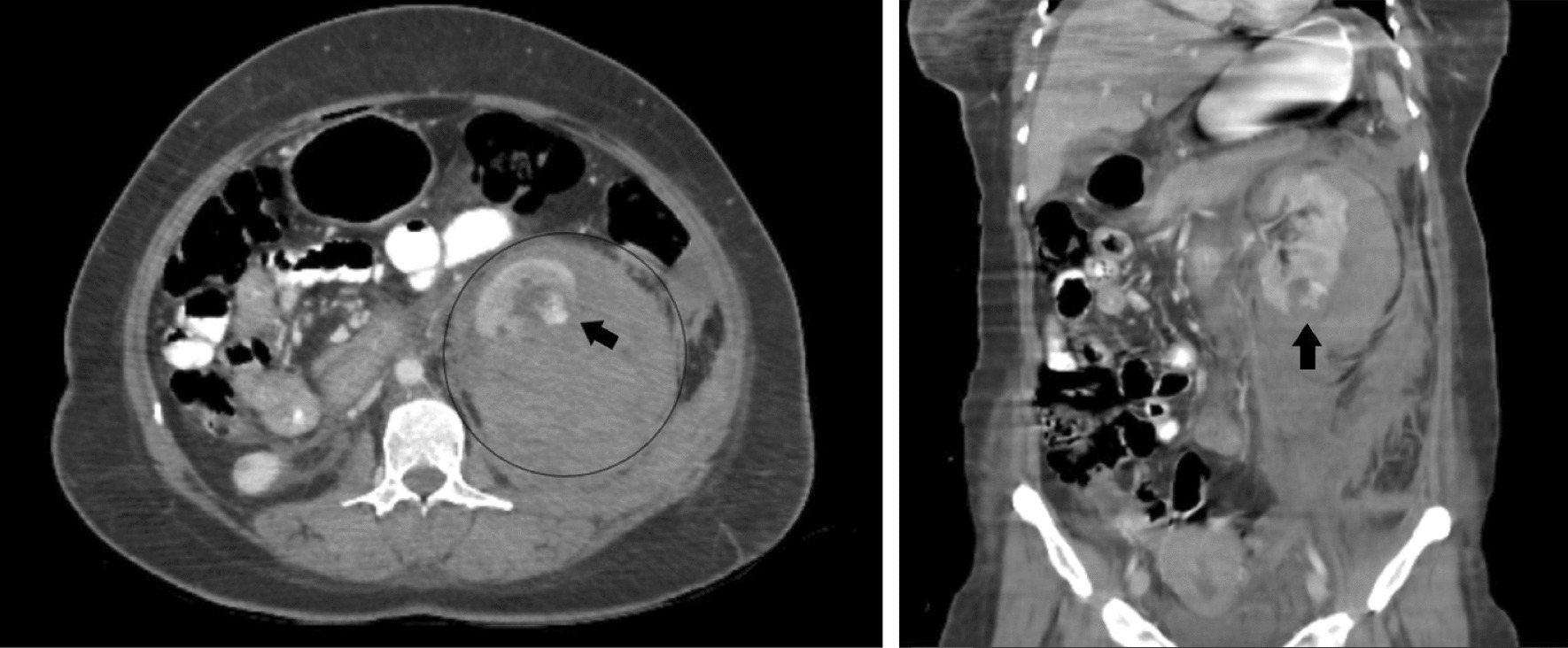
Abdominopelvic computed tomography scan with intravenous contrast in the axial (left) and coronal (right) planes. A hypodense lesion (black arrow) with foci of intense enhancement and active bleeding was identified in the lower pole of the left kidney. Additionally, the lesion was surrounded by a hematoma (black circle) with a pressure effect, leading to an anterior displacement of the left kidney

Considering the patient’s clinical presentation, an abdominopelvic ultrasound was conducted in the emergency department. The results demonstrated an empty uterine cavity, accompanied by a hypoechoic lesion within the lower pole of the left kidney. Furthermore, moderate fluid collection was identified in the left pararenal and perisplenic spaces. Subsequently, an abdominopelvic computed tomography (CT) scan with intravenous contrast (arterial phase) identified a hypodense lesion (30 × 25 × 20 mm^3^) with mild heterogeneous enhancement in the lower pole of the left kidney. Hyperdense foci within the renal mass with CT number of 160 Hounsfield Units (HU) on arterial phase images were suggestive of active bleeding. In addition, a hematoma (130 × 108 × 90 mm^3^) was observed in the subcapsular, perinephric, and anterior and posterior pararenal spaces. This hematoma caused anterior displacement of the left kidney, attributed to pressure effects (Fig. 2). Moreover, findings regarding the endometrial cavity and myometrium were unremarkable except for a defect in the superior segment of the uterine anterior wall. Furthermore, owing to the observation of pleural effusion in the limited view of the thorax in the abdominopelvic CT scan, the patient underwent a chest CT scan, which disclosed bilateral pleural effusion, more prominent on the left side, with adjacent passive lung collapse. No evidence of pulmonary nodules or masses was detected.

Considering the enhancing hemorrhagic lesion within the left kidney, spontaneous renal hemorrhage (SRH) with potential underlying causes, including renal cell carcinoma, metastatic lesion, and angiomyolipoma, was proposed. In light of the patient’s previous history of complete molar pregnancy with inadequate follow-up and a high serum β-hCG level, renal metastasis from choriocarcinoma was considered. As a result, a laparotomy exploration was planned on the basis of the patient’s clinical condition and the surgical team’s preference in our center. During the laparotomy, an extensive left retroperitoneal hematoma was discovered. Additionally, a lacerated mass was actively bleeding at the lower pole of the left kidney. Consequently, a partial nephrectomy was performed using the wedge resection technique with a 5-mm margin. Further exploration of the intraperitoneal cavity revealed a left para-ovarian cyst without evidence of an ectopic pregnancy. Thus, a cystectomy was carried out. The patient also underwent endometrial curettage. Throughout the surgery, all specimens were documented for pathology evaluation.

**Fig. 3:**
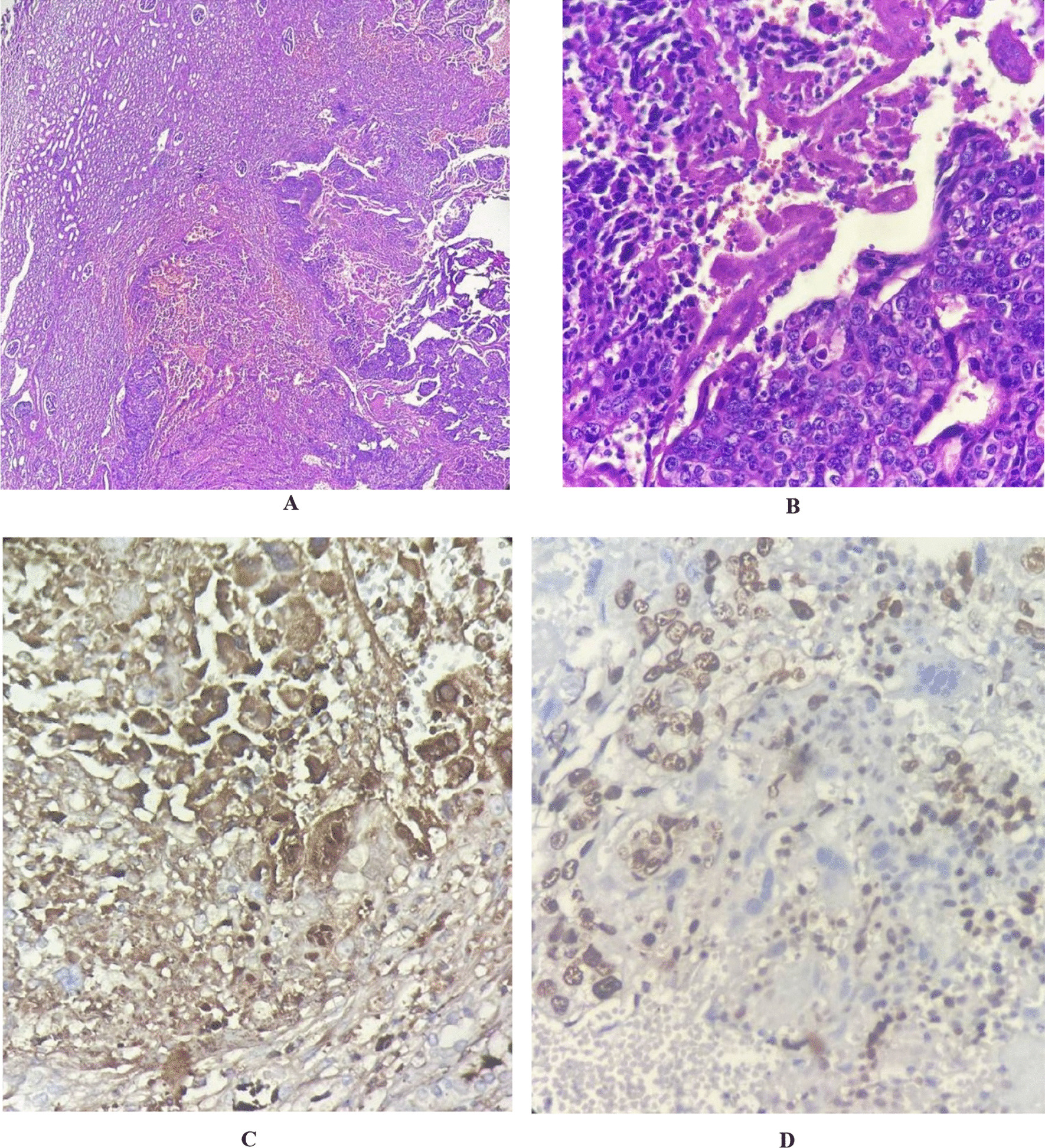
Histopathology of metastatic choriocarcinoma with adjacent renal tissue. **A** (10 × magnification) and **B** (40 × magnification) showed evidence of marked nuclear atypia with hemorrhage, necrosis, and mitotic figures (hematoxylin and eosin staining). In addition, tumoral cells were immunohistochemically positive for beta human chorionic gonadotropin hormone (**C**) and Ki 67 (**D**)

The histopathological findings of the renal mass strongly aligned with characteristic features of choriocarcinoma, including a disorganized mixture of various types of trophoblast cells, significant cytologic pleomorphism, nuclear enlargement, extensive mitotic activity, a hemorrhagic or necrotic background along with viable tumor cells at the periphery, and lymphovascular tumor thrombi [15]. Moreover, the immunohistochemical analysis revealed that tumoral cells were strongly positive for β-hCG, cytokeratin 7, and Ki 67. In this regard, among all gestational trophoblastic neoplasia, only invasive mole and choriocarcinoma demonstrate a high expression of β-hCG, serving as a pivotal biomarker [6]. Furthermore, considering choriocarcinoma as a hyperproliferative tumor, the high expression of Ki 67, an index for mitotic activity and proliferation, is a distinctive feature of choriocarcinoma [6]. In light of these findings, the histopathology results confirmed the final diagnosis as choriocarcinoma renal metastasis (Fig. 3). Notably, no malignant cells were observed in endometrial curettage specimens, and a corpus luteum cyst was identified within the left para-ovarian cyst.

After the surgery, although the patient did not experience complications associated with central nervous system involvement, she underwent brain magnetic resonance imaging with gadolinium for precise staging before initiating chemotherapy. The results indicated no pathological enhancement or metastatic lesions. Additionally, a thoracentesis was performed, revealing the absence of malignant cells in the pleural fluid cytology.

Taken together, these findings clarified that the patient had high-risk gestational trophoblastic neoplasia (stage IV:11 according to The International Federation of Gynecology and Obstetrics (FIGO)/World Health Organization (WHO) staging and scoring system [1]). Owing to the choriocarcinoma histological diagnosis, immediate initiation of chemotherapy was planned. A multi-agent regimen was selected to prevent drug resistance [16]. Considering the acceptable rate of complete response and long-term survival, as well as the lower toxicity of the etoposide, methotrexate, actinomycin D, cyclophosphamide, and vincristine/oncovin (EMA-CO) regimen [16], this regimen was preferred. The patient was referred to the oncology department to receive a standard EMA-CO regimen. After receiving chemotherapy courses for 3 weeks, the serum β-hCG level decreased to 5 mIU/mL.

After being discharged from the hospital, the patient has been undergoing monthly measurements of β-hCG level while on hormonal contraception. Over the past 6 months of follow-up, the β-hCG levels have consistently remained within the normal ranges, showing no evidence of relapsing disease or new-site metastasis. With regard to the rarity of our case and the highest risk of recurrence within the first year of treatment cessation among patients with high-risk gestational trophoblastic neoplasia [17], we planned to continue monthly monitoring of β-hCG levels for at least 1 year in this patient.

## Discussion and conclusion

In this report, we describe a rare case of post molar choriocarcinoma with a solitary left renal metastasis in the absence of a primary uterine tumor that presented with pure urological symptoms alongside SRH and was successfully treated with a combination of partial nephrectomy and systemic chemotherapy.

Considering the rarity of choriocarcinoma renal metastasis, in reviewing the available literature, we found only nine publications addressing post molar choriocarcinoma with renal involvement, as detailed in Table 1.

**Table 1:** Characteristics of previous reports describing post molar choriocarcinoma with renal metastasis

Number	Report (year)	Age (years)	Clinical manifestation	Childbearing history	Hydatiform mole treatment	Interval	Primary uterine tumor	Site of metastasis	Surgical treatment	Chemotherapy
1	Kutcher *et al.* [8] 1977	29	Hematuria	Hydatiform mole	Spontaneous abortion Chemotherapy	1 year	1.5 cm choriocarcinoma nodule within the myometrium	Unilateral kidney Lung Liver pelvis	Hysterectomy	Regimen not reported
2	Thanikasalam *et al.* [9] (1991)	52	Hemoptysis Dyspnea Chest pain	Gravida 11 Para 11 Hydatiform mole	Hysterectomy Bilateral salpingo-oophorectomy	2 years and 9 months	–	Unilateral kidney Lung Liver	–	Five courses of triple regimen: Methotrexate Actinomycin Etoposide
3	Ikeda *et al.* [10] (1996)	34	Gross hematuria Loin pain Fever	Hydatiform mole	Dilatation and suction evacuation	2 years	Without primary tumor	Unilateral kidney Lung	Nephrectomy Total hysterectomy	Seven courses of EMA-CO regimen
4	Karadeniz *et al.* [5] (2011)	50	Weakness Right lumbar pain Painless total hematuria Weight loss	Term pregnancy Menopause	Hysterectomy Bilateral salpingo-oophorectomy	5 years	–	Bilateral kidneys Lung Liver Spleen	Unilateral transperitoneal radical nephrectomy	Methotrexate-based chemotherapy
5	Yen *et al.* [11] (2019)	50	Headache Dyspnea Hemoptysis	Gravida 1, para 1 Menopause Hydatiform mole	Hysterectomy	10 years	–	Bilateral kidneys Lung Brain Liver Duodenum Urinary bladder	-	One course of BEP Omitting bleomycin owing to poor pulmonary function Three courses of cisplatin and etoposide Initiation of EMA-CO regimen owing to residual tumor and elevated β-hCG
6	Pietrus *et al.* [3] (2021)	37	Lumbar pain Hematuria Hemoptysis Fever Skin lesion Temporary vision loss	Miscarriage Hydatiform mole	Hysterectomy Unilateral salpingo-oophorectomy	6 years	–	Bilateral kidneys Lung Skin Brain	–	Induction phase: EP Continuation and consolidation phase: EMA-CO for 12 months Including TP owing to chemotherapy resistance after 12 months
7	Mustafa *et al.* [12] (2021)	33	Loss of consciousness Left pupil fixed dilation Vaginal bleeding	Ectopic pregnancy Miscarriage Hydatiform mole	Not reported	Not reported	A well-defined mass located at the anterior wall of the uterus	Unilateral kidney Brain Pleura Ovary with invasion to the bladder Small intestines	Brain hematoma evacuation Hysterectomy Bilateral oophorectomy Unilateral radical nephrectomy Small intestinal metastasis excision	Regimen not reported
8	Zribi *et al.* [13] (2021)	52	Headache One episode of seizure	Menopause Hydatiform mole	Dilatation and suction evacuation	8 years	Without primary tumor	Unilateral kidney Brain Spleen Lung	Whole brain radiotherapy Splenic artery embolization	Induction phase: etoposide and cisplatin Continuation and consolidation phase: EMA-CO
9	Pahwa *et al.* [14] (2023)	29	Abdominal pain	Hydatiform mole	Dilatation and curettage	10 years	Without primary tumor	Unilateral kidney lung	Segmentectomy of lung lesion Radical nephrectomy	EMA-CO regimen

EMA-CO, etoposide, methotrexate, actinomycin D, cyclophosphamide, and vincristine/oncovin; BEP, bleomycin/etoposide/cisplatin; β-hCG, beta human chorionic gonadotropin hormone; EP, etoposide and cisplatin; TP, paclitaxel/cisplatin

In the reported cases, the patients’ ages ranged from 29 to 52 years, with a mean of 40.66 years. The mean interval between the antecedent hydatiform mole and the occurrence of choriocarcinoma was approximately 5.5 years (ranging from 1 to 10 years). Notably, three patients were diagnosed with post-menopausal choriocarcinoma [5, 11, 13]. Choriocarcinoma presented various clinical symptoms correlated with metastasis sites. The most commonly reported clinical presentations included hematuria, hemoptysis, and site-specific pain. In most cases, renal metastases were identified through imaging evaluations, except in the reports by Kutcher *et al.* [8], Ikeda *et al.* [10], and Karadeniz *et al.* [5]. Remarkably, all cases described synchronous metastases, with the most frequent sites being the lung, brain, and liver. Bilateral kidney involvement was documented in only three patients [3, 5, 10]. Four patients experienced the development of post molar choriocarcinoma after undergoing hysterectomy for antecedent hydatiform mole [3, 5, 9, 11]. Excluding patients with a history of hysterectomy, the primary uterine tumor was mentioned in only the two reports by Kutcher *et al.* [8] and Mustafa *et al.* [12]. Finally, all patients received chemotherapy, and most of them underwent appropriate surgical treatments depending on the locations of their metastases.

Reviewing these reports has highlighted a diagnostic dilemma concerning post molar choriocarcinoma. Owing to its rarity and diverse presentations, gynecologic history, especially the antecedent molar pregnancy, was not comprehensively evaluated initially in most cases. Additionally, β-hCG level was not measured upon admission [3, 5, 10–12, 14], even among women of reproductive age [3, 10, 12, 14]. In these instances, β-hCG level was assessed following choriocarcinoma diagnosis during histopathological evaluation. Furthermore, despite patients’ undergoing appropriate surgical treatment and chemotherapy, there exists no consensus on selecting chemotherapy regimens, chemotherapy duration, or surgical approaches. The details of chemotherapy regimens and duration, as well as surgical procedures, are available in Table 1.

In our case, choriocarcinoma renal metastasis was detected in the absence of a primary tumor within the uterus. Similarly, several previous reports have described extrauterine metastases from post molar choriocarcinoma without a uterine primary tumor [10, 13, 14]. In contrast, Mustafa *et al.* [12] and Kutcher *et al.* [8] reported cases with a primary tumor of post molar choriocarcinoma within the uterus. Considering the undetectable choriocarcinoma primary tumor within the uterus, the extrauterine involvement could be attributed to a primary uterine neoplasm that underwent spontaneous necrosis and complete regression [18, 19].

Regarding choriocarcinoma renal metastasis as an arterial metastasis, it has been suggested that renal involvement may result from circulating tumor cells originating from initial lung metastases [20]. In line with this, all reports of post molar choriocarcinoma outlined in Table 1 have delineated the renal involvement with coexistent lung metastasis, except for a report by Mustafe *et al.* [12], which described multi-organ metastases, including a single kidney, in the absence of lung metastasis. Correspondingly, in the current case, solitary kidney involvement occurred without lung metastasis.

Renal metastasis from choriocarcinoma can present with symptoms such as hematuria, flank or abdominal pain, oliguria, or SRH. However, it is important to note that most cases of renal involvement are incidentally diagnosed during imaging studies [5, 20]. To our best knowledge, only one prior report has described post molar choriocarcinoma with renal metastasis resulting in SRH. Pahwa *et al.* [14] documented the case of a 29-year-old woman complaining of abdominal pain who was diagnosed with choriocarcinoma and had renal and lung metastasis in the absence of a primary uterine tumor a decade after experiencing a complete hydatiform mole. The patient underwent a segmentectomy for the lung mass and a radical nephrectomy, which revealed bleeding and a retroperitoneal hematoma. Afterward, she received EMA-CO chemotherapy, and there was no evidence of recurrence during the 6-month follow-up period. In contrast to the study by Pahwa *et al.*, the patient in our case was older and highly symptomatic. Choriocarcinoma developed 2 years after a complete molar pregnancy, and only a partial nephrectomy was performed owing to solitary renal metastasis. Similar to the report by Pahwa *et al.*, there was no primary tumor identified within the uterus in our case. The patient received the EMA-CO regimen, and no evidence of recurrence was observed during the 6-month follow-up.

SRH is characterized by the acute development of nontraumatic subcapsular and perirenal hematomas with various underlying causes [21]. Renal neoplasms, such as angiomyolipoma, renal cell carcinoma, and metastatic involvement, are recognized as the most common causes of SRH [19, 21]. Considering the diagnostic challenges for SRH etiologies, renal metastasis from choriocarcinoma might be an unusual etiology of SRH among females of childbearing age, especially those with history of molar pregnancy [2, 7, 19]. Choriocarcinoma bleeding tendency could be attributed to the capability of trophoblastic tumors to invade and damage vessel walls, as well as the presence of fragile vessels within the tumors [2, 12]. In many cases, renal involvement by choriocarcinoma has been misdiagnosed as renal malignancies before histopathology confirmation. Therefore, screening β-hCG levels is recommended to exclude the possibility of renal metastasis from choriocarcinoma among young women with urological symptoms and suspected renal masses [4].

As shown in Table 1, previous reports have documented renal involvement in post molar choriocarcinoma alongside other common sites of metastases. As far as we are aware, the current report presents the first case of post molar choriocarcinoma with solitary renal metastasis, without the presence of primary uterine tumor or metastases in other sites, which presented with urological symptoms and SRH. Considering this notable strength, we did not employ DNA analysis to confirm the origin of the renal metastasis as gestational choriocarcinoma. However, several factors supported our clinical diagnosis, including the patient’s age, a prior complete molar pregnancy with inadequate follow-up, a 2-year interval between events, an elevated β-hCG level, histopathological confirmation, and a significant reduction in β-hCG owing to chemotherapy [2, 5, 7].

In summary, although renal involvement by gestational choriocarcinoma is rare, this study highlights that choriocarcinoma with solitary renal metastasis could present with only urological symptoms in the absence of a primary uterine tumor or metastasis to other sites. In other words, nongynecological symptoms can serve as the initial presentations of metastatic choriocarcinoma, and the hemorrhagic nature of choriocarcinoma may result in an acute and rapid onset event. Therefore, measuring β-hCG levels might play a crucial role in evaluating reproductive-aged females experiencing persistent and unexplained nongynecological symptoms, such as hematuria or SRH, particularly in those with history of antecedent molar pregnancy. Furthermore, integrating various imaging modalities provides comprehensive information for diagnosing metastatic choriocarcinoma and guides the selection of an appropriate treatment approach. Considering the choriocarcinoma’s excellent response to chemotherapy, immediate initiation of chemotherapy can be life-saving, and monitoring complete disease remission would be easily accessible through regular measurement of β-hCG levels.

## Data Availability

All data and materials are available from the corresponding author upon request.

## Abbreviations

β-hCG: Beta human chorionic gonadotropin hormone
SRH: Spontaneous renal hemorrhage
GTD: Gestational trophoblastic disease
CT: Computed tomography
HU: Hounsfield units

## References

[1] Ngan HY, Seckl MJ, Berkowitz RS, Xiang Y, Golfier F, Sekharan PK, Lurain JR, Massuger L (2018). Update on the diagnosis and management of gestational trophoblastic disease. Int J Gynecol Obstet.

[2] Vijay R, Kaduthodil M, Bottomley J, Abdi S (2008). Metastatic gestational trophoblastic tumour presenting as spontaneous subcapsular renal haematoma. Br J Radiol.

[3] Pietrus M, Czekaj A, Dziadkowiak D, Ratajczyk K, Trzciniecki M, Kowal P, Mytsyk Y (2021). Metastatic choriocarcinoma presenting as renal colic and skin lesion–case report. Wiad Lek.

[4] Du Y, Zhang X, Sun S, Sun M, Yang D, Yu X, Li K, Ma J, Li Y, Ge J (2022). Case Report: 18F-FDG PET/CT and laparoscopic nephron sparing surgery in the management of bleeding from renal metastases of choriocarcinoma. Front Oncol.

[5] Karadeniz T, Topsakal M, Ozkaptan O, Cakır C (2011). Bilateral renal choriocarcinoma in a postmenopausal woman. Korean J Urol.

[6] da Silva ALM, de Nascimento Monteiro K, Sun SY, Borbely AU (2021). Gestational trophoblastic neoplasia: novelties and challenges. Placenta.

[7] Mangla M, Singla D, Kaur H, Sharma S (2017). Unusual clinical presentations of choriocarcinoma: a systematic review of case reports. Taiwan J Obstet Gynecol.

[8] Kutcher R, Lu T, Gordon DH, Becker JA (1977). Renal choriocarcinoma metastasis: a vascular lesion. AJR Am J Roentgenol.

[9] Thanikasalam K (1991). Post-hysterectomy choriocarcinoma with pulmonary and renal metastases. Med J Malaysia.

[10] Ikeda I, Miura T, Kondo I, Kimura A (1996). Metastatic choriocarcinoma of the kidney discovered by refractory hematuria. Acta Urologica Japonica.

[11] Yen C-C, Tsai H-W, Yen C-J (2019). p metastatic choriocarcinoma mimicking primary lung cancer. J Cancer Res Pract.

[12] Mustafa MK, Matti WE, Kadhum HJ, Alsubaihawi ZA, Kareem ZM, Al-Sharshahi ZF, Hoz SS. Spontaneous intracerebral haemorrhage as an initial presentation of a choriocarcinoma: a case report. Romanian Neurosurg. 2021:48–51.

[13] Zribi A, Al Mazroui R, Sayani R, Burney IA. An Unusual Presentation of Choriocarcinoma in A postmenopausal woman: a case report. Sultan Qaboos Univ Med J. 2021. 10.18295/squmj.5.2023.036 PMC10906773 38434469

[14] Pahwa S, Sharma A, Kamboj M, Gupta G, Pasricha S (2023). Metastatic choriocarcinoma of the kidney in the absence of existing primary uterine tumor: a rare presentation. J Cancer Res Ther.

[15] Hui P (2019). Gestational trophoblastic tumors: a timely review of diagnostic pathology. Arch Pathol Lab Med.

[16] Braga A, Elias KM, Horowitz NS, Berkowitz RS (2021). Treatment of high-risk gestational trophoblastic neoplasia and chemoresistance/relapsed disease. Best Pract Res Clin Obstet Gynaecol.

[17] Balachandran K, Salawu A, Ghorani E, Kaur B, Sebire NJ, Short D, Harvey R, Hancock B, Tidy J, Singh K (2019). When to stop human chorionic gonadotrophin (hCG) surveillance after treatment with chemotherapy for gestational trophoblastic neoplasia (GTN): a national analysis on over 4,000 patients. Gynecol Oncol.

[18] Li H-M, Hou W-C, Lai Y-J, Kao C-C, Chao T-K, Yu M-H, Su H-Y (2016). Gestational choriocarcinoma with renal and pulmonary metastases lacking a primary uterine origin. Taiwan J Obstet Gynecol.

[19] Lal A, Singhal M, Kumar S, Bag S, Singh S, Khandelwal N (2009). Bilateral renal and jejunal metastasis of choriocarcinoma presenting as spontaneous renal hemorrhage. Cancer Imaging.

[20] Wang Y-e, Song H-z, Yang X-y, Dong S-y, Gan N (1991). Renal metastases of choriocarcinoma: a clinicopathological study of 31 cases. Chin Med J.

[21] Ahn T, Roberts MJ, Navaratnam A, Chung E, Wood S (2017). Changing etiology and management patterns for spontaneous renal hemorrhage: a systematic review of contemporary series. Int Urol Nephrol.

